# Primary Effects of Intravitreal Bevacizumab in Patients with Diabetic Macular Edema

**DOI:** 10.12669/pjms.294.3735

**Published:** 2013

**Authors:** Iftikhar-ul-Haq Tareen, Azizur Rahman, P.S Mahar, Muhammad Saleh Memon

**Affiliations:** 1 Dr. Iftikhar-ul-Haq Tareen, FCPS,; 2 Prof. Azizur Rahman, MCPS, FCPS,; 3 Prof. P.S Mahar, FRCS, FRCOphth,; 4 Prof. Muhammad Saleh Memon, DO, FRCS,

**Keywords:** Best Corrected Visual Acuity (BCVA), Central Macular Thickness (CMT), Diabetic Macular Edema (DME), Intra Vitreal Bevacizumab (IVB)

## Abstract

***Objective:*** To evaluate the efficacy of primary intra vitreal bevacizumab (IVB) injection on macular edema in diabetic patients with improvement in best corrected visual acuity (BCVA) and central macular thickness (CMT) on optical coherence tomography (OCT).

***Methods:*** This prospective interventional case series study was conducted at Retina Clinic, Al-Ibrahim Eye Hospital, and Isra Postgraduate Institute of Ophthalmology Karachi. Between December 2010 to June 2012. BCVA measurement with Early Treatment in Diabetic Retinopathy Study (ETDRS) charts and ophthalmic examination, including Slit-lamp bio microscopy, indirect ophthalmoscopy, Fundus fluorescein angiography (FFA) and OCT were done at the base line examination. At monthly interval all patients were treated with 3 injections of 0.05 ml intra vitreal injection containing 1.25 mg bevacizumab. Patients were followed up for 6 months and BCVA and OCT were taken at the final visit at 6 month.

***Results:*** The mean BCVA at base line was 0.42±0.14 Log Mar units. This improved to 0.34±0.13, 0.25±0.12, 0.17±0.12 and 0.16±0.14 Log Mar units at 1 month after 1^st^, 2^nd ^3^rd^ injections and at final visit at 6 months respectively, a difference that was statistically significant (P>0.0001) from base line. The mean 1mm CMT measurement was 452.9 ± 143.1 µm at base line, improving to 279.8 ± 65.2 µm (P<0.0001) on final visit. No serious complications were observed.

***Conclusions:*** Primary IVB at a dose of 1.25 mg on monthly interval seems to provide stability and improvement in BCVA and CMT in patient with DME.

## INTRODUCTION

With the increasing prevalence of diabetes in the world wide, Diabetic retinopathy (DR) remains the major threat to sight in the working-age population. It remains as a major cause of blindness in developing countries.^1^ According to the Diabetic Association of Pakistan – World Health Organization (DAP-WHO) survey (1994-1998), overall prevalence of Diabetes is 11.47% in Pakistani population.^2^ Another study has revealed that 25% of those patients, that present to the health care facilities in Pakistan with diabetes suffered from retinal complications.^3^ The Wisconsin study^4^ reported that 9% of diabetic population had macular edema with in 1 disc diameter of the fovea. A study carried out at Al Ibrahim Eye Hospital (2011) showed that 39.8% of patients registered at retinal clinic were suffering from DR, out of which 45% were having Clinically Significant Macular Edema (CSME).^5^

Retinal hypoxia is the primary cause of DR which increases the expression of vascular endothelial growth factor (VEGF). It is an endothelial cell-specific mitogen which induces angiogenesis and increase vascular permeability by affecting endothelial tight-junction protein.^6^ In ocular vascular disease such as DME, VEGF levels has been found considerably higher in macular region.^7^ The Early Treatment Diabetic Retinopathy (ETDRS) shows 3-year risk of moderate visual loss due to macular edema was 32%, and focal macular laser photocoagulation was effective in the treatment of DME. ETDRS demonstrated that immediate focal laser photocoagulation reduced the risk of moderate visual loss by 50% (from 24% to 12%, 3 year after initiation of treatment). However, 12% of treated eyes still lost ≥ 15 ETDRS letters at 3-year follow-up interval. Furthermore, only 3% of laser-treated eyes experienced a gain of ≥ 3 lines of vision.^8^ The failure of laser photocoagulation in these eyes has prompted interest in other treatment modalities such as Anti-VEGF agents^9^, intravitreal triemcinolon acetonide (IVTA),^10^ pars plana vitrectomy (PPV)^11^ or treatment with protein Kinase C inhibitors.^12^

Food and Drug Administration (FDA) has approved bevacizumab (Avastin, Genentech Inc. South San Francisco, CA, USA) a fusion protein with human antibody backbone against VEGF, it binds and inhibits all the active forms of VEGF and is used in the treatment of metastatic colorectal cancer. Some studies had found it useful in the reduction of macular edema secondary to central retinal vein Occlusion (CRVO),^13^ vascular permeability and neovascularization secondary to age-related macular degeneration (AMD).^14^

The purpose of this study was to evaluate the best corrected visual acuity (BCVA) measured on ETDRS chart and central macular thickness (CMT) carried out on optical coherence tomography (OCT) after a series of 3 injection of IVB at interval of one month and at mean follow up of 6 months after 1^st^ injection.

## METHODS

All patients were followed up for 6 months. Approval of the study was obtained from the institutional ethical committee, and informed consent was obtained from all patients. The study followed the principles of Declaration of Helsinki. 

Patients with evidence of DME were included in this study. DME is defined as the evidence of diffuse retinal thickening, hard exudates (with out a circinate ring pattern) involving the center of the macula (clinically significant diabetic macular edema as defined by ETDRS), or both and Diffuse fluorescin leakage involving the center of the macula on FFA, and Central Macular Thickening (CMT) on Optical Coherence Tomography (OCT).

The exclusion criteria included patients with DME who had been treated previously with laser photocoagulation, intra vitreal triamcinolone or any Anti-VEGF therapy elsewhere. The patients with Macular ischemia, uncontrolled intraocular pressure, intra ocular surgery within past 6 months, history of vitreo retinal surgery of the study eye, or any evidence of epiretinal membrane or vitreomacular traction were excluded.

After detailed history each patient underwent baseline examination which included BCVA measurement with ETDRS charts and ophthalmic examination, including slit lamp bio microscopy and dilated fundus examination with +90 diopter lens and indirect ophthalmoscopy. All patients had fundus fluorescien angiography (FFA).Baseline CMT was analyzed by OCT using 6 diagonal slow 6mm radial line scan on Topcon analyzer (Tokyo, Japan).

***Procedure: ***All eyes had several drops of local anesthetic (Alcaine – Alcon, Belgium) with dilatation of pupil (Mydriacyl 1% - Alcon, Belgium) before the procedure. After the eye had been prepped in standard fashion using 5% povidoniodine, an eye lid speculum was used to stabilize the eyelids and the injection of 1.25 mg (0.05 ml), bevacizumab was performed at 3.5 to 4mm posterior to the limbus through the inferiotemporal pars plana with a tuberculin syringe and 30 gauge needle After injection, retinal artery perfusion was checked with the indirect ophthalmoscopy. A single drop of antibiotic (Vigamox – Alcon, Belgium) is instilled and patients were instructed to administer topical antibiotics for one week.

All patients were examined at one week and one month after the first injection. At 1^st^ month visit BCVA was recorded and 2^nd^ injection was administered and the same was repeated after one month and the 3^rd^ injection was administered .The patients were followed-up for 6 months and final BCVA and CMT were taken at the final visit at 6 month after the first injection.

## RESULTS

A total of 49 diabetic patients were enrolled in this study. Seven of our patients were lost the follow-up or were excluded due to exclusion criteria from the study. The final data of 78 eyes of 42 patients with a minimum follow up of 6 months after 1^st^ injection of bevacizumab were included for analysis. The patients had a mean age of 53.5 ± 11.4 years, and 25(59.52%) were male, 17(40.47%) were female.

Within one month after the initial bevacizumab injection improvements in BCVA were observed, and these significant changes continued throughout the 6 months follow up period. The mean BCVA at baseline was 0.42 ± 0.14 Log Mar units. At one month after the 1^st^ injection BCVA improved to 0.34±0.13 Log Mar unit, a difference that was statistically significant (P<0.0001). This improvement in BCVA was maintained after 2^nd^ and 3^rd^ injections which were 0.25±0.12 Log Mar units and 0.17±0.12 Log Mar units respectively. In addition the mean BCVA at 6 month follow up examination was 20/25 (0.16±0.14 Log Mar units), a statistically significant difference from baseline BCVA (P<0.0001).(Table-I)

Further subgroups analysis demonstrated that after 1^st^ injection, 49(62.8%) eyes improved one or more ETDRS line of BCVA,27(34.6%) eyes remained stable and 2(2.5%) decreased one or more ETDRS lines of BCVA. This trend continued after 2^nd^ and 3^rd^ injection, and at 6 month follow up examination, 16(20.5%) eyes remained stable, 57(73.0%) eyes improved one or more ETDRS lines and 5(6.4%) eyes had decreased one or more ETDRS lines of BCVA. (Table-II)

**Fig.1:**
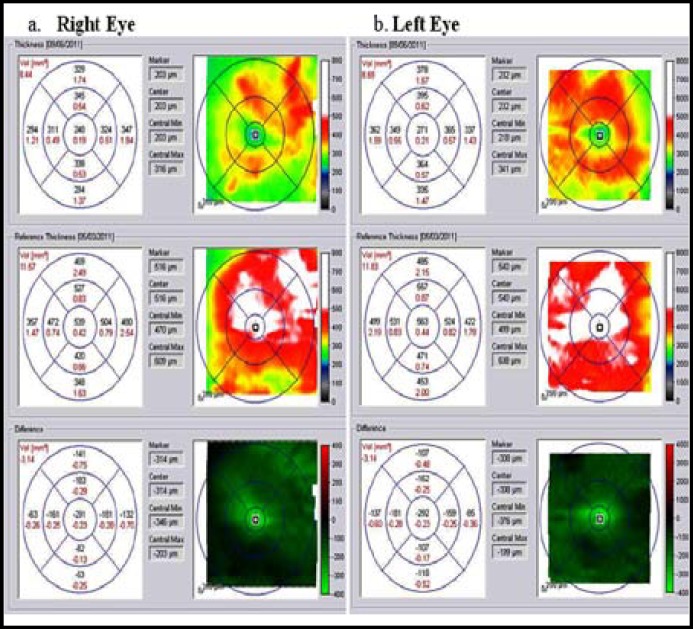
Optical Coherence tomography (OCT) imaging of a 52 years old diabetic male with a history of loss of vision in both eyes to 20/160, Right eye (a) Left eye (b), in which diabetic macular edema had developed. The OCT shows a marked resolution in macular edema and improvement of Central Macular Thickness (CMT) after treatment

Optical Coherence tomography results were available for all 78 eyes at baseline and at 6 months follow up examination. At baseline the mean 1-mm CMT measurements was 452.9±143.1 µm, improving to 279.8±65.2 µm at 6 month follow up examination, which was significantly different from baseline (P<0.0001).(Fig.1)

A mild anterior chamber cellular reaction was observed in 9 (11.5%) eyes, but the condition improved with use of topical corticosteroid and antibiotic combination drops (Tobradex – Alcon, Belgium). No other injection or drug related complications were observed.

**Table-I:** Mean Log Mar value for the BCVA 1 month after each injection and at 6 month follow up examination

*BCVA*	*Mean Log Mar Unit*	*Std Deviation *	*Snellen equivalent*
At baseline	0.42	±0.14	20/50
After 1^st^ injection	0.34	±0.13	20/40
After 2^nd^ injection	0.25	±0.12	20/30
After 3^rd^ injection	0.17	±0.12	20/25
At 6 month Follow up	0.16	±0.14	20/25

**Table-II:** Best Corrected Visual Acuity (BCVA) analysis by subgroups (78 eyes

	*After 1* ^st^ * Injection*		*After 2* ^nd^ * Injection*		*After 3* ^rd^ * injection*		*At 6 month follow up*	
	*No of eyes*	*Percentage*	*No of eyes*	*Percentage*	*No of eyes*	*Percentage*	*No of eyes*	*Percentage*
Improved ≥ 1 ETDRS lines	49	62.8%	53	67.9%	54	69.2%	57	73.0%
Remained stable	27	34.6%	22	28.2%	21	26.9%	16	20.5%
Decreased ≥ 1 ETDRS lines	2	2.5%	3	3.8%	3	3.8%	5	6.4%

**Fig.2:**
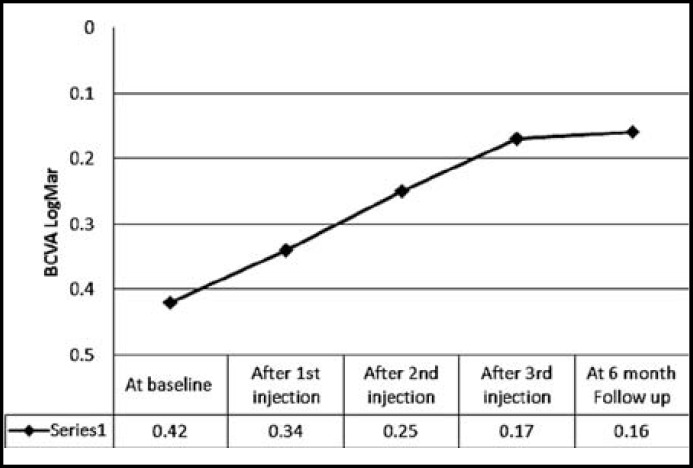
Changes in best corrected visual acuity (BCVA) after intravitrealbevacizumab (IVB). BCVA improved after 1^st^ injection from 0.42 Log Mar (baseline) to 0.34 Log Mar, a difference that was statistically significant (P<.0001), this improvement of BCVA was maintained throughout after 2^nd^ and 3^rd^ injections and final visit at 6 months

## DISCUSSION

This study reports on 78 consecutive eyes with DME treated with Intravitreal Bevacizumab, which resulted in both anatomic and functional improvement. In most of patients improvement of BCVA were detected with in the first month after the 1^st^ injection and continued throughout the study period. (Fig.2)

The exact pathophysiologic mechanism responsible for DME remains uncertain, retinal hypoxia and various rheological disturbances play a role in the disruption of the inner blood-retinal barrier associated with metabolic alterations.^6^ The long term circulatory disturbance may lead to functional vascular obstruction, relative retinal ischemia, and release of cytokines such as VEGF. Funatsuet al^7^ reported that level of VEGF was elevated in vitreous fluid of subjects with DME. VEGF causes conformational changes in the tight junctions of retinal endothelium and plays a major role in increasing vascular permeability in the progression of DME.

Several treatment methods for DME are under investigation. ETDRS has demonstrated that, the risk of visual loss from DME can be reduced by laser photocoagulation.^8^ However, in some eyes macular edema may persist despite laser photocoagulation. Therefore clinicians had developed interests in other treatment methods such as pharmacologic therapies. Intravitreal triamicinilone acetonide (IVTA) injection has some promising results but this treatment modality is not without its risks and complication.^10^ These complications were related to the injection procedure or to the corticosteroid suspension.^15^ Oral protein Kinase C inhibitors had demonstrated efficacy in prevention of visual loss from DME.^12^ Currently Fluocinolon Acetonide vitreous inserts are under investigations showing some promising results.^16^

The important role of VEGF in the breakdown of the blood-retinal barrier and vascular permeability resulting in retinal edema is clear. Therefore, the rationale for use of anti-VEGF agents for treatment of DME is significant. Bevacizumab (Avastin; Genentech, Inc) is a fusion protein with human antibody backbone that binds to all sub types of VEGF and used successfully in tumor therapy as a systemic drug. Recently off label use of IVB injection has been presented in small cohorts of patients with macular edema from various causes like CRVO^13^, AMD^14^ and Proliferative Diabetic Retinopathy (PDR).^17^ As VEGF disrupts the inner blood-retinal barrier and causes exravasation of fluids by affecting endothelial tight junctions. Therefore, VEGF inhibition by IVB has been emerged as a target molecule for the treatment of DME.

There are several studies in the literature on the intravitreal administration of anti-VEGF for DME. Recently Khan et al^18^ published a report of 26 eyes, followed up for 3 months after IVB injection. The mean BCVA at baseline 0.726 Log Mar was improved to 0.452 Log Mar at 3rd month. Flourescien leakage stopped in 25 (96.15%) eyes. Kumar and Sinah^19^ reported results of 20 eyes with DDME treated with IVB at a dose of 1.25mg that had not responded to previous photocoagulation. They concluded that IVB resulted in a significant decrease in macular thickness and improvement in BCVA at 3 month. Ozkiris A^20^ presented results of 30 eyes, out of which 24 (80%) eyes showed increased visual acuity after mean, follow up time of 5.6 months after IVB injection. BCVA improved from 1.09±0.23 Log Mar (at baseline) to 0.90±0.17 Log Mar at 1^st^ month and 0.77±0.26 Log Mar at the last visit. The mean edema map values significantly decreased by 33.3%. Arevalo et al^21^ published a report of 24-month anatomic and visual acuity response after primary intravitreal bevacizumab in patients with Diffuse Diabetic Macular Edema (DDME). They compared 2 different doses of intravitreal bevacizumab, 1.25mg and 2.5 mg respectively. They found that primary IVB at doses of 1.25 or 2.5 mg seem to provide stability and improvement in BCVA, OCT, and FA results in DME at 24 months.

The results of our study compare favorably with these reports and confirm their findings. In our study, we found that improvement in BCVA was achieved within a month after 1^st^ IVB injection. A further improvement was achieved after each injection of bevacizumab and was maintained at 6 months follow up. BCVA in our study improved from 0.42±0.14 Log Mar unit at base line to mean 0.34±0.13 Log Mar unit after one month of 1^st^ IVB injection and further improvement to 0.25±0.12 Log Mar unit after one month of 2^nd^ injection and this trend continued to the final visit at 6 month with BCVA improving to 0.16±0.14 Log Mar unit. The mean central 1mm CMT improved from 452.9±143.1 µm at base line to 279.8±65.2 µm at 6 month visit. This confirms the finding of previous studies which shows that the eyes with DME treated with IVB result in both anatomic and functional improvement.

The results of our and other above mentioned studies indicate that IVB injection may have a beneficial effect on macular thickness and BCVA in DME. In future this new treatment method may replace or compliment focal or grid laser photocoagulation.

Our study has some limitations. First, the follow up time was relatively short, but visual and anatomical responses were apparent during the follow-up time. Second, there is no control group in this study as randomization was not possible. Third, this study was carried out in a single center so results cannot be generalized.

## CONCLUSION

This study demonstrated that IVB injection is an effective approach with promising results for the primary treatment of DME. IVB at a dose of 1.25mg on at least monthly interval seems to provide stability and improvement in BCVA and CMT in DME at 6 moths. However evaluation in a multicenter, randomized, controlled clinical trial comparing IVB with other treatment modalities is needed to evaluate the safety and efficacy of this treatment method.

## Authors Contribution:

IT: Conceived and designed the protocol, did data collection and statistical analysis, manuscript writing and editing.

AR: was involved in clinical management of patients.

P.SM and SM: Critically reviewed the manuscript for final publication.
